# Impact of patient education meetings on disease knowledge in chronic urticaria patients

**DOI:** 10.55730/1300-0144.6107

**Published:** 2025-11-04

**Authors:** Nazan BEYHAN, Hatice Eylül BOZKURT YILMAZ, Aslı ÇİFTCİ, Ömür AYDIN, Dilşad MUNGAN, Betül Ayşe SİN, Gülfem Elif ÇELİK, Murat TÜRK, İnsu YILMAZ, Sevim BAVBEK

**Affiliations:** 1 Division of Immunology and Allergy, Department of Pulmonary Diseases, Faculty of Medicine, Ankara University, Ankara, Turkiye; 2 Division of Immunology and Allergy, Department of Pulmonary Diseases, Faculty of Medicine, Erciyes University, Kayseri, Turkiye; 3 Department of Biostatistics, Faculty of Medicine, Ankara University, Ankara, Turkiye

**Keywords:** Chronic urticaria, angioedema, disease knowledge, healthcare outcomes, patient management, education meetings

## Abstract

**Background/aim:**

Chronic urticaria (CU) is a manageable disease with symptoms of itching and visible lesions that can disrupt daily life and reduce quality of life. The severity of symptoms can vary, and the cause is often unknown. Concerns among patients as regards to treatment and prognosis highlight the need for disease education to enhance self-management and reduce anxiety. The present study examines the effect of disease education on the knowledge level of CU patients.

**Materials and methods:**

Patients with CU who presented to the allergy clinics of two tertiary university hospitals were invited to in-person education sessions including slide presentations given by faculty members. A 27-question survey was administered before and after the sessions addressing the subject matter, including definitions, causes, diagnostic tests, and treatment options for CU.

**Results:**

Included in the study were 83 patients (57 female; 26 male). The average number of correct answers prior to the education session was 13.62, and this increased to 19.48 after education. The most frequently incorrect answers were related to the importance of skin prick tests in cases of urticaria, the daily doses of antihistamines, and the use of topical corticosteroid treatment. The average number of correct answers post-education increased significantly among the university graduates, from 15.45 to 21.72 (p < 0.001), in high school graduates from 11.15 to 17.78 (p < 0.001), and in middle school graduates from 11.71 to 18.14 (p = 0.018). In contrast, the increase was not statistically significant among primary school graduates (p = 0.252). A total of 77 (92%) patients increased their scores after the education session.

**Conclusion:**

Patients can benefit from the provision of accurate and reliable information about their disease. Face-to-face education effectively increased knowledge across all educational levels in patients with CU, and reduced their reliance on unreliable online sources. This enhanced understanding is expected to lead to better treatment adherence and self-management skills.

## 1. Introduction

Chronic urticaria (CU) is a chronic disease characterized by recurrent wheals, angioedema (AE), or both, persisting for more than 6 weeks [1–3]. The disease causes intense itching, redness, and in cases involving AE in particular, can significantly alter the patient’s physical appearance, affect virtually every aspect of daily life, and result in a profound deterioration in quality of life [4,5]. This burden is exacerbated in cases of high disease activity or frequent AE episodes [6]. Despite treatment with high-dose H1-antihistamines, the disease remains uncontrolled in 41.9% of patients, resulting in frustration with therapy and uncertainty about the future [7,8].

Chronic urticaria not only affects individual health but also has significant societal and economic burdens [9]. Patients often get caught up in a persistent search to identify the cause of their condition, as well as its course, triggers, and potential treatment options [10]. This uncertainty-driven pursuit frequently results in unnecessary repeat healthcare visits and costly diagnostic tests [11]. Such factors contribute to increased healthcare expenditures and productivity losses, highlighting a need to address the disease from a broader perspective. In this context, developing an effective patient education program for patients holds significant potential in CU management. Therapeutic patient education (TPE) has been successfully utilized for over 3 decades to support the management of chronic diseases, and to enhance clinical outcomes, promote treatment adherence, and improve communication between patients and physicians [12]. While the effectiveness of TPE has been well-documented in chronic diseases such as diabetes, hypertension, and asthma, studies into its application in CU are limited [13].

The present study addresses this critical gap in the literature concerning the management of CU through patient education. CU was selected as the model disease for this intervention due to its status as a chronic condition with fluctuating symptoms requiring long-term self-management, and the frequent misconceptions regarding its etiology and treatment, making patient education particularly relevant. CU is one of the most frequently encountered conditions in our Immunology and Allergy Department and Center of Urticaria Excellence. The primary aim of the present study is to assess the efficacy of education and to identify gaps and deficiencies in patient knowledge with a view to developing a reproducible educational model for potential future implementation. As one of the few studies to date focusing on patient education in CU, this research provides evidence supporting the integration of structured education into routine clinical care.

## 2. Materials and methods

This prospective cross-sectional study was conducted between 2023 and 2024 at the Immunology and Allergy Diseases Clinics of Ankara University Faculty of Medicine and Erciyes University Faculty of Medicine. Included in the study were patients diagnosed with CU according to the EAACI/GA2LEN/WAO criteria. All patients over the age of 18 years were invited to participate in face-to-face educational sessions, while those with acute urticaria, those under 18 years of age, and those with mental or cognitive impairments that could prevent them from completing the survey were excluded from the study. All patients who consented to participate and who provided written informed consent were included in the study prior to enrollment. The study was approved by the Ankara University Ethics Committee (Approval No: 2023/361, Date: August 10, 2023).

**Figure 1:**
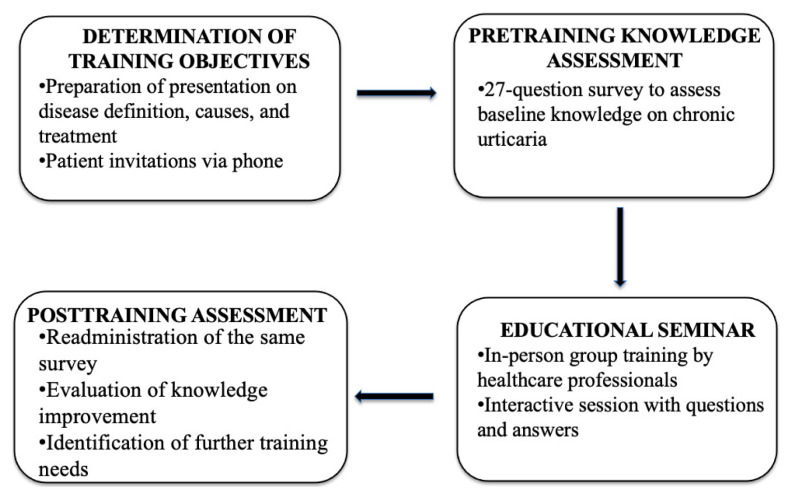
Flowchart of the overall study design and implementation process.

A standardized PowerPoint presentation was delivered to the groups by clinical faculty members, supported by visual materials. The overall training process is summarized in the flowchart in Figure 1. Each session included a structured lecture on CU, followed by an interactive question-and-answer segment. The educational content was developed by a multidisciplinary team of allergy specialists and medical educators, paying heed to international guidelines and patient-identified needs. The curriculum comprehensively addressed key clinical aspects of disease understanding and management.

The program covered the definition and pathophysiology of CU, underlying etiological factors, and common triggers, with particular emphasis on comorbidities such as thyroid disease. The limited role of allergens in the development of the disease was also clarified, and information was provided on the diagnostic process and the appropriate use of diagnostic tests. The correct use and dosage of antihistamines, as well as second-line treatment options, were explained in detail. Finally, the influence of stress and lifestyle-related triggers was discussed, and common myths related to diet and allergies were raised and clarified.

To assess baseline and post-intervention knowledge, a 27-item questionnaire developed by the study team was administered to the participants before and after the sessions. The questionnaire was based on existing guidelines, current literature, and expert input, and included close-ended statements with three response options (“Agree”, “Disagree”, and “Don’t Know”). The content domains included disease definition, etiological factors, comorbidities (e.g., thyroid disease), the role of allergens, stress and other triggers, diagnostic methods, antihistamine dosages, and additional therapies (Supplementary File).

Because the questionnaire had not been validated in previous studies, it underwent face and content validation by an expert panel prior to use. All responses were scored, and correct answers were aggregated to yield a total knowledge score. No predefined threshold was set for “adequate knowledge”, however, a comparative analysis of the pre- and postintervention scores was used to evaluate educational impact.

Data were analyzed using SPSS version 11.5. Statistical analyses included the Wilcoxon signed-rank test, chi-square test, and Spearman correlation analysis. Statistical significance was set at p < 0.05.

## 3. Results

A total of 95 patients were initially evaluated, and 83 were included in the final study after 12 were excluded as they did not meet the inclusion criteria. The mean age of the final sample was 43.52 ± 13.23 years, the mean disease duration was 6.7 ± 7.7 years (range: 1–40 years), and 48% of the patients were university graduates. The most common comorbid diseases were hypertension (15.7%), allergic rhinitis (14.5%), and diabetes mellitus (14.5%). Among the patients, 28 (33.7%) were receiving antihistamine therapy, three (3.6%) were receiving omalizumab, and 52 (62.7%) were receiving both treatments concurrently. The demographic data of the patients are summarized in Table.

**Figure 2:**
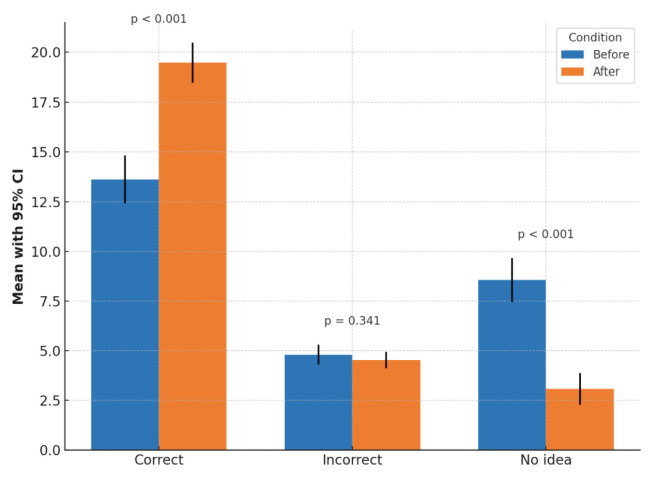
Bar chart showing the mean values and 95% confidence intervals for correct, incorrect, and no idea responses before and after training.

**Figure 3:**
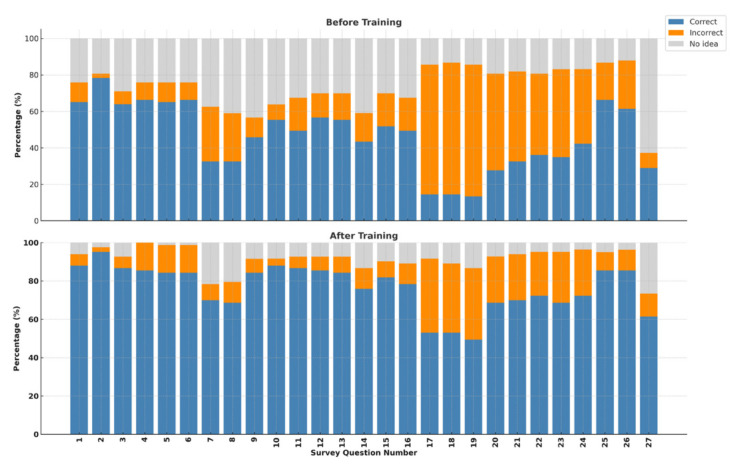
Distribution of correct, incorrect, and no idea responses to each survey question before and after training.

The education sessions significantly improved the knowledge of the participants, with the mean correct responses increasing from 13.62 in the pretest to 19.48 after the educational sessions (p < 0.001). While incorrect responses decreased from slightly 4.80 to 4.53, the change was not statistically significant (p = 0.341). The most notable improvement was noted in the “I don’t know”, responses, which dropped significantly from 8.56 to 3.08 (p < 0.001) (Figure 2). The average pre- and posttraining results for each question are presented in Figure 3.

Prior to the educational sessions, the mean number of correct responses among the omalizumab users was 14.41, compared to 12.07 in the nonomalizumab group (p = 0.064). The mean number of incorrect responses among omalizumab users was 3.07, compared to 5.21 for nonomalizumab users, and the difference was statistically significant (p = 0.014). The mean number of “I don’t know” responses was 10.96 in the omalizumab user group and 7.34 in the nonomalizumab group; and the difference was not statistically significant (p = 0.087).

After the educational sessions, no statistically significant differences were observed between the omalizumab users and nonusers in terms of correct (p = 0.985), incorrect (p = 0.521), or “I don’t know” (p = 0.727) responses.

The most frequently incorrectly answered questions before and after the education sessions were related to whether a skin prick test was always necessary in urticaria, the maximum daily dosage of antihistamines, and the role of steroid creams in treatment. Before the educational sessions, the majority of patients (37.3%, n = 31) were unsure whether urticaria was a chronic disease, and 25 patients (30.1%) answered this question incorrectly. In response to the question, “Should every urticaria patient follow an allergen-restricted diet?” 39.8% (n = 33) of the participants answered incorrectly, and 36.1% gave no opinion. The question “Can urticaria be triggered by stress?” was the most correctly answered question, both before (86.7%) and after (91.6%) the educational sessions.

When the impact of educational status on the responses of the participants was analyzed, it was observed that 44.4% of primary school graduates, 28.6% of middle school graduates, 21% of high school graduates, and 72.9% of university graduates provided responses above the pre-training median of 13 correct answers.

**Figure 4:**
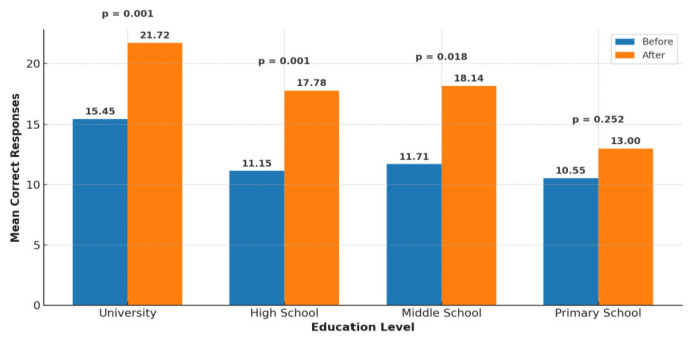
Change in the average number of correct answers to 27 questions among patients before and after training according to educational status.

After the educational sessions, the average number of correct answers increased significantly from 15.45 to 21.72 among university graduates (p < 0.001), from 11.15 to 17.78 among high school graduates (p < 0.001), and from 11.71 to 18.14 among middle school graduates (p = 0.018). Among the primary school graduates, the increase from 10.55 to 13.00 was not statistically significant (p = 0.252) (Figure 4).

Statistical analyses revealed no significant differences in pre-education knowledge levels based on disease duration or sex (p > 0.05 for all comparisons), and similarly, post-education knowledge scores were not significantly affected by disease duration or sex. In contrast, a statistically significant difference was noted in correct post-education scores associated with age, as participants younger than the mean age (≤43 years) recorded a mean post-education correct score of 21.12 ± 3.11, whereas those aged >43 years had a mean score of 18.07 ± 5.82 (p = 0.025, Mann–Whitney U test). These findings suggest that the younger participants benefited more from the educational intervention than their older counterparts.

**Figure 5:**
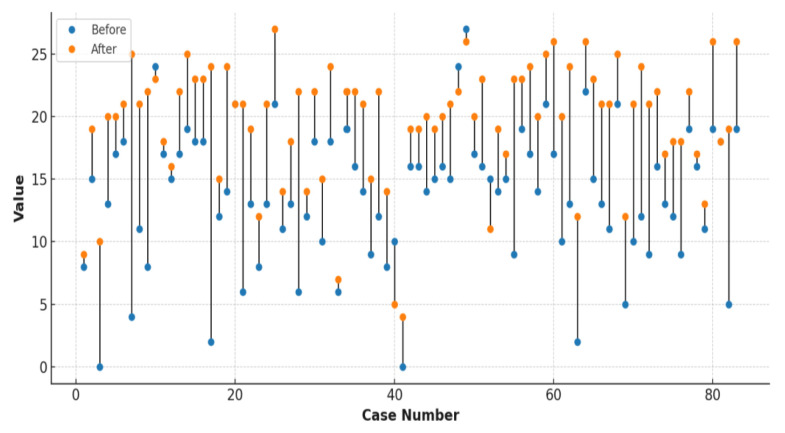
Changes in the number of correct answers to the 27 survey questions, comparing the pre- and posttraining results for 83 patients. A significant increase in the total number of correct answers was observed in 77 patients after training.

Overall, 77 (92%) patients saw an increase in the number of correct answers following the education sessions (Figure 5).

## 4. Discussion

Our study evaluated the impact of face-to-face education programs on the knowledge levels of patients with CU. To the best of our knowledge, this is the first multicenter study in Türkiye conducted to systematically evaluate structured patient education specifically for patients with CU, providing a valuable contribution to the limited literature in this field. A significant increase in patient knowledge was observed after the training program, and the improvement was more pronounced among those with a higher educational status. The increase in knowledge among patients with primary and middle school education, in contrast, was limited, indicating a greater need for education in this group. While the training programs improved the patients’ awareness of the causes, triggers, and management strategies of the disease, some misconceptions and attitudes remained unchanged. In particular, more comprehensive information needs to be provided on topics such as allergies, diet, and treatment practices. It was noted that the patients had a high level of awareness regarding the impact of stress on the disease.

It is apparent from the study findings that patients need more information about their disease, including its causes, progression, triggering factors, available treatment options, and prognosis. In a study involving 1800 participants investigating the use of information and communication technologies such as web browsers, YouTube, and social media for the dissemination of health-related information, it was found that 99.6% of patients had used at least one such platform [14]. Among CU patients, X (formerly Twitter), the web, and Instagram are stated to be the most frequently used platforms for obtaining disease-related information [10]. It should be noted, however, that social media and Internet search engines can lead to inaccurate or misleading health information [15]. Many videos are misleading, of low quality, uploaded by unidentified individuals, and some even promote unproven alternative treatments [16]. Face-to-face education provided to patients by healthcare workers can serve as a reliable source of accurate information, enabling patients to share their experiences, find social support, and better manage their conditions. It was observed in the preset study that the majority of patients increased their knowledge of their condition following the face-to-face patient educational sessions.

University graduates were the most successful group in our survey. As seen in other chronic disease models [17,18], higher education levels lead to a better understanding of the condition, while contributing to medication adherence and clinical outcomes. While the level of knowledge increased significantly among middle school, high school, and university graduates after the provided education, the knowledge level of primary school graduates did not increase to the desired extent following urticaria education. It is thought that a higher level of education contributes to a better understanding and management of the disease by enhancing health literacy [17]. In a previous study, education level was found to be significantly associated with disease knowledge, attitudes to health, and practical skills, with individuals with higher education levels contributing to a better understanding and management of the disease through increased health literacy [18]. Patients with a higher education status have are better equipped to utilize health information effectively, while those with lower education levels require more intensive educational support. We believe that patients with lower education levels would benefit from more frequent and prolonged face-to-face education sessions to improve their understanding and management of their disease. Addressing educational inequalities through accessible, simplified, and repetitive education sessions is essential if equitable knowledge gains across all patient groups are to be obtained. The use of simplified language, visual materials, and example-based instructional techniques may enhance the effectiveness of education, particularly among older adults and patients with limited health literacy.

The question most accurately answered by the patients related to the association between disease and stress. The relationship between chronic urticaria and stress is well known [19]. Patients in our study cohort had a high level of awareness of this association. The burden of CU is not limited to a decline in quality of life, as there is also an additional strain on the healthcare system due to frequent visits to healthcare providers [20]. At the onset of symptoms, patients often seek information online or attempt self-treatment with over-the-counter medications before consulting a doctor [14]. However, when these methods fail, they enter a cycle of repeated doctor or clinic visits [21]. Complex care processes may hinder the access of patients to effective medical services, while time constraints can prevent doctors from providing sufficient information to their patients [18]. These challenges lead to patient dissatisfaction, and contribute to anxiety and depression, when compared to the general population [22]. Interactive educational programs between patients and nurses have been reported to help bridge information gaps, reduce the prevalence of anxiety and depression, and positively impact quality of life and patient adherence [23]. In CU patients, in whom psychological comorbidities are common, incorporating psychosocial support into the patient education programs provided by physicians can play a critical role in improving both the physical and psychological health outcomes.

In the present study, the average duration of urticaria was 6.7 years, highlighting the chronic nature of the disease in a significant portion of patients. This chronicity likely contributes to psychological distress and fatigue, as supported by previous studies linking longer disease duration with reduced adherence and impaired quality of life [22]. These findings underscore the need for early, sustained, and psychosocially informed educational strategies, particularly for patients experiencing prolonged symptoms.

Despite attending the education sessions, many of the CU patients in our study were still under the misapprehension that their condition is related primarily to allergies, leading them to undergo frequent allergy tests. This is a common misconception; in reality, allergies only rarely cause CU [1]. Diagnostic tests, such as skin evaluations for aeroallergens or food allergens should only be conducted when strongly indicated by the patient’s clinical history [24,25]. Although a low-histamine diet is not a standard treatment for all CU patients, identifying the small subset of patients who may benefit from such dietary adjustments should be a priority in education programs [25,26]. Given that allergies only rarely cause CU, it is essential to clarify to patients that comprehensive allergy testing and food elimination are not necessary in most cases, and that dietary changes should be reserved for carefully selected patients. In short, effective patient education is crucial for correcting misconceptions on this matter and ensuring proper management.

In the test conducted before the educational program, a considerable number of patients answered the question about the daily maximum dose of antihistamines incorrectly. Surprisingly, this misunderstanding persisted even after training, suggesting that an adequate level of understanding had not been achieved. This may be related to the fact that standard-dose antihistamine therapy was sufficient for disease control in a significant portion of patients, or that some patients perceived increasing the dose up to four times the standard dose as unnecessary or excessive. One of the most frequently misunderstood factors related to the use of topical corticosteroids in CU treatment. While oral corticosteroids can be used when necessary, topical corticosteroids and topical antihistamines have no role in CU management [27].

For the first- and second-line treatment of CU, second-generation H1-antihistamines are recommended at licensed or increased doses (up to four times the standard dose) [3]. However, these treatments fail to provide adequate control in approximately half of all CU patients [28]. Our results echo this challenge, as knowledge gaps related to dose escalation remained following the educational sessions. It is apparent that this is one of the main reasons for poor treatment adherence among CU patients. This is supported by a study in which 72% of the patients failed to adhere to the recommended treatment regimens [29].

It is clear that providing patients with regular self-management education, particularly regarding dose escalation, is of critical importance. Self-management education has proven to be effective for the improvement of treatment outcomes and quality of life, and for reducing depression and anxiety [30,31]. Written action plans that include dose escalation of antihistamines and the addition of oral steroids when necessary could increase patient knowledge, reduce unplanned clinic and emergency room visits, and contribute to better management.

Although clinical outcomes such as disease activity reduction, frequency of relapses, or improvement in quality of life were not directly assessed in the present study, previous evidence from chronic disease models suggests that patient knowledge is strongly correlated with better self-management and reduced symptom burden [30,31]. It is thus reasonable to expect that the significant improvement in knowledge observed in our study may potentially translate into fewer disease flares, enhanced treatment adherence, and improved patient satisfaction. Future longitudinal studies should be conducted to validate this hypothesis and to assess the impact of educational interventions on clinical outcomes in CU patients.

The education of patients with urticaria can help them understand the causes and triggers of the condition, and to manage their condition more effectively. Such education should emphasize symptom management, the correct use of medications, and the importance of treatment adherence, while also providing strategies to cope with depression and anxiety. Such a process will foster effective communication between the patient and their physician, encourage active participation in treatment, and improve adherence. Additionally, patients who learn how to act correctly in crisis situations can reduce unnecessary emergency room visits.

Through the provision of education, patients can be better informed about their symptoms and treatment options, and the impacts of urticaria, empowering them to better manage their condition [32]. Our study has aimed to bridge the gap in patient-physician interactions by providing up-to-date information garnered from experts in the field, while alleviating the challenges faced by individuals with urticaria. Face-to-face education serves as a valuable support system, contributing to effective disease management and improvements in quality of life.

Patients with chronic conditions tend to communicate with others with similar conditions, sharing their experiences and providing social support. We recommend the organization of active patient education programs in all allergy and immunology centers on October 1st, World Urticaria Day, as well as collaborations with patient associations. Patient education helps individuals manage their condition better, improve adherence to treatment, and enhance their quality of life. In addition to traditional face-to-face education, the use of digital tools such as video-based training, online seminars, and mobile applications should also be considered, as this may improve the accessibility of patients with lower levels of education.

The limitations of this study include the absence of a control group, the lack of assessment of long-term knowledge retention, the small sample size, and the failure to evaluate the impact of education on the Urticaria Control Test (UCT) and Urticaria Activity Score (UAS). That said, its multicenter design enhances the generalizability of the findings, and the use of a standardized training program ensures consistency in knowledge delivery. Changes in knowledge levels were thoroughly evaluated through structured surveys, and the significant increase in correct response rates following education underscored the effectiveness of the program. Future studies should aim to assess the long-term impact of educational interventions on medication adherence, quality of life, disease activity, healthcare resource utilization, and patient behavior.

## 5. Conclusion and recommendations

Increasing the knowledge levels of patients through education will enhance treatment adherence and self-management skills. Improving the knowledge of patients about their disease will contribute to better symptom control, fewer flares, reduced healthcare visits, and an overall improvement in quality of life. Ensuring patients remain informed will contribute to their psychological well-being. This highlights the critical role of patient education as an adjunct to pharmacologic treatment in the comprehensive management of CU.

Studies of this patient group are essential due to the chronic nature of the condition. Adherence to, and continuity of treatment are key to effective management. Therefore, regular education courses on the causes, triggers, treatment options, and emergency action plans can be expected to improve treatment success.

Future studies should evaluate whether the knowledge improvements demonstrated in this study lead to sustained clinical benefits, including reduced urticaria activity, improved UCT and UAS outcomes, and decreased healthcare resource utilization. In addition, exploring the effectiveness of digital and hybrid education tools, particularly for patients with low health literacy, may offer new avenues for accessibility and effectiveness of patient education programs.

## Supplementary File

Dear Patient,

We kindly ask you to complete this questionnaire regarding your urticaria/angioedema condition. By completing the questionnaire before and after the presentation, we aim to increase your knowledge about the disease and hope to receive valuable feedback to improve the educational content.

Thank you for your participation.

Full Name: ...............................................................................................................................................................................................

Age: ...........................................................................................................................................................................................................

Sex: □ Female □ Male

Education Level: □ No formal education □ Primary School □ Middle School □ High School □ University

Please indicate if you have any other allergic diseases besides urticaria (asthma, hay fever, drug allergy, food allergy, bee allergy, allergic eczema, allergic shock (anaphylaxis), etc.): ...................................................................................................................................................................................................................

Please indicate any other chronic diseases you have (hypertension, diabetes, thyroid disorder, heart disease, liver disease, kidney disease, migraine, depression, etc.): ...................................................................................................................................................................................................................

For how many years have you had urticaria? .....................................................................................................................................

Please list the names of the medications you use for urticaria: ...................................................................................................................................................................................................................

Please answer the following questions by marking (x) in the appropriate box:

**Table:** 

Statement	Agree	Disagree	No idea/Don’t know
1. Urticaria is a skin disease.			
2. Another name for urticaria is hives or nettle rash.			
3. Urticaria occurs only in adults.			
4. It is generally benign.			
5. In urticaria, redness and swelling may disappear and reappear within a day.			
6. Infectious diseases can cause urticaria.			
7. Urticaria is a permanent disease.			
8. It can be seen together with thyroid disease.			
9. Allergy causes urticaria in only a small number of patients.			
10. The cause of urticaria is mostly unknown.			
11. Physical factors (heat, cold, pressure, vibration) can trigger urticaria.			
12. Every urticaria patient must follow an allergen-free diet.			
13. Certain foods can aggravate urticaria.			
14. Cat and dog allergies can cause urticaria.			
15. Certain medications can worsen urticaria.			
16. Urticaria plaques are seen only on the trunk.			
17. Imaging tests such as X-ray or CT are used in urticaria.			
18. Allergy testing is always required in urticaria.			
19. Urticaria improves when the allergen is avoided.			
20. Oral medications (antihistamines) are used in the treatment of urticaria.			
21. Antihistamines (Zyrtec, Allerset, Bilaxten, Aerius, Deloady, Fexadyne, Rupafin, Kestine, etc.) can be taken at most twice a day.			
22. Cortisone creams are used in the treatment of urticaria.			
23. Injectable medications (omalizumab) are used when urticaria cannot be controlled with drugs.			
24. Urticaria can be triggered by stress.			
25. Sometimes swelling (angioedema) may occur without urticaria, affecting the lips, face, eyelids, throat.			
26. Angioedema may be hereditary.			
27. Hereditary angioedema does not respond to standard treatments and requires special therapy.			

## Figures and Tables

**Table:** Demographic characteristics of patients with chronic urticaria (n = 83).

Variable	n	%
**Sex**		
Female	57	68.7
Male	26	31.3
**Age (years) mean ± SD**	43.52 ± 13.23	
**Educational status**		
Primary school	9	10.8
Middle school	7	8.4
High school	19	22.9
University	48	57.8
**Comorbid diseases**		
Asthma	6	7.2
Rhinitis	12	14.5
Eczema	5	6.0
Drug allergy	10	12.0
Food allergy	3	3.6
Migraine	8	9.6
Diabetes mellitus	12	14.5
Hypertension	13	15.7
Thyroid diseases	10	12.0
**Mean disease duration (years)**	6.7 ± 7.7	
**Treatment received**		
Antihistamine	28	33.7
Omalizumab	3	3.6
Antihistamine + omalizumab	52	62.7

n: number of patients; SD: standard deviation.
